# Comprehensive transcriptional analysis reveals salt stress-regulated key pathways, hub genes and time-specific responsive gene categories in common bermudagrass (*Cynodon dactylon* (L.) Pers.) roots

**DOI:** 10.1186/s12870-021-02939-1

**Published:** 2021-04-10

**Authors:** An Shao, Wei Wang, Shugao Fan, Xiao Xu, Yanling Yin, Amombo Erick, Xiaoning Li, Guangyang Wang, Hongli Wang, Jinmin Fu

**Affiliations:** Coastal Salinity Tolerant Grass Engineering and Technology Research Center, Ludong University, Yantai, Shandong People’s Republic of China

**Keywords:** Common bermudagrass, Root, Transcriptome analysis, WGCNA, Hub genes, Time-specific response

## Abstract

**Background:**

Despite its good salt-tolerance level, key genes and pathways involved with temporal salt response of common bermudagrass (*Cynodon dactylon* (L.) Pers.) have not been explored. Therefore, in this study, to understand the underlying regulatory mechanism following the different period of salt exposure, a comprehensive transcriptome analysis of the bermudagrass roots was conducted.

**Results:**

The transcripts regulated after 1 h, 6 h, or 24 h of hydroponic exposure to 200 mM NaCl in the roots of bermudagrass were investigated. Dataset series analysis revealed 16 distinct temporal salt-responsive expression profiles. Enrichment analysis identified potentially important salt responsive genes belonging to specific categories, such as hormonal metabolism, secondary metabolism, misc., cell wall, transcription factors and genes encoded a series of transporters. Weighted gene co-expression network analysis (WGCNA) revealed that lavenderblush2 and brown4 modules were significantly positively correlated with the proline content and peroxidase activity and hub genes within these two modules were further determined. Besides, after 1 h of salt treatment, genes belonging to categories such as signalling receptor kinase, transcription factors, tetrapyrrole synthesis and lipid metabolism were immediately and exclusively up-enriched compared to the subsequent time points, which indicated fast-acting and immediate physiological responses. Genes involved in secondary metabolite biosynthesis such as simple phenols, glucosinolates, isoflavones and tocopherol biosynthesis were exclusively up-regulated after 24 h of salt treatment, suggesting a slightly slower reaction of metabolic adjustment.

**Conclusion:**

Here, we revealed salt-responsive genes belonging to categories that were commonly or differentially expressed in short-term salt stress, suggesting possible adaptive salt response mechanisms in roots. Also, the distinctive salt-response pathways and potential salt-tolerant hub genes investigated can provide useful future references to explore the molecular mechanisms of bermudagrass.

**Supplementary Information:**

The online version contains supplementary material available at 10.1186/s12870-021-02939-1.

## Background

Soil salinity is a significant abiotic factor limiting plant growth and development. To mitigate salt-induced osmotic stress, ion toxicity and oxidative damage, plants have evolved a series of physiological and molecular response mechanisms [1–3]. Common bermudagrass (*Cynodon dactylon* (L.) Pers.) is a popular and extensively used turf species which can be spread by stolons, rhizomes, and seed [4, 5]. Despite having good salt tolerance level, there is a wide intraspecies variation. Thus, the growth and development of relatively sensitive cultivars could be seriously inhibited by salinity stress, greatly limiting the promotion and application of bermudagrass in saline soils [6, 7]. Therefore, an in-depth analysis of salt tolerance mechanism and mining key response genes and pathways will contribute to its application in saline environments.

In plants, salt stress triggers a genome-wide transcriptomic reprogramming to response to this environmental stimuli. As a result, groups of genes related to many physiological traits and salt-response pathways are regulated to alleviate the adverse effects, making salt response to be a complex quantitative trait [1, 8]. Immediately after plants perceive salt stress signal from the environment, multiple signal transduction pathways can be rapidly activated [9, 10] and an elevation in the calcium ion (Ca^2+^) concentration is among the first response to external stimuli [11]. To cope with the stress, the action of stimuli Ca^2+^ sensors (e.g., CBLs: calcineurin B-like proteins; CIPKs: Ca^2+^-independent protein kinases; CDPKs: Ca^2+^-dependent protein kinases; CMLs: calmodulin-like proteins) [11] precedes a chain of reactions such as the SOS (salt overly-sensitive) and MAPK (mitogen-activated protein kinase) pathways [12–14]. Along Ca^2+^ signalling, other second messengers such as ROS (reactive oxygen species) are also participated [3]. Although ROS can function as signalling molecules in response to environmental cues [2, 15], its excessive accumulation can result in cell oxidative damage [16]. To curb ROS-induced oxidative damage, plants have evolved a complex scavenging system consisting of antioxidants enzyme (e.g. superoxide dismutase, SOD; peroxidase, POD; catalase, CAT) and non-enzymatic scavengers (e.g. tocopherols; carotenoids; phenols) have been developed to scavenge excessively produced ROS and protect themselves from salt-induced oxidative stress [17, 18]. Also, the phytohormones-mediated signalling pathways (e.g. auxin; abscisic acid, ABA; jasmonic acid, JA; cytokinin, CTK; gibberellin, GA; ethylene, ETH) also play key roles in the adaptive growth of plants after environmental stimulation [19].

To further protect plants from damage, activated cascades such as Ca^2+^, ROS, and hormone signaling cascades can further activate other regulators like transcription factors (TFs) (e.g. ABA-responsive element-binding protein/ABA-binding factor, ABRE/ABF) [20] to regulate other downstream salt response genes. For instance, genes regulating levels of osmoprotectants are reported to be the first stress-inducible transcripts during initial response to initial osmotic stress. The intracellular concentrations of osmolytes such as proline, soluble sugar and dehydrins are elevated to improve cellular osmotic pressure [2, 21]. After prolonged exposure to salt stress, other strategies are adopted to mitigate Na^+^ toxicity (24 h or beyond), for example, the ion transporters such as HKT (high-affinity K^+^ transporter) and NHX (Na^+^/H^+^ antiporters) gene families could be regulated to further sequester or compartmentalize excess Na^+^ in the vacuole with a higher cytosolic K^+^/Na^+^ and resist to salt stress in glycophytes [22, 23]. In soybean, after treated for 24 h or beyond, the seedlings entered a new physiological state with lower photosynthetic rates and stomatal conductance, followed by the accumulation of Na^+^ in the leaf that could be detrimental to the plants. Therefore, 24 h might be a turning point at which salt response strategy might begin to change in many plants [24].

Under excessive salt exposure, roots are the first organs to detect the stress and likely to suffer more damage due to their closer proximity compared to the shoots [25, 26]. As a result, roots perceive an early-onset osmotic stress and respond. Subsequently, these initial reactions can be passed on to the whole plant [2]. This makes roots to be ideal for providing a sensitive target to study the molecular mechanisms underlying plant salt tolerance and adaptation [27]. In other species, several salt-responsive transcriptomic studies in the roots have been done so far [24, 28, 29]. In bermudagrass roots, using two cultivars with contrasting salt tolerance level, a transcriptome analysis was performed after 7 days of salt stress [30]. However, transcriptomic studies in the roots of bermudagrass that involve in early salt response among multi-time points have not been explored. Taking in consideration these temporal dynamic changes when evaluating a plant’s response to a stress factor could provide a more systematic analysis in the expression profiles [25, 28, 29]. In this study, we investigated and compared gene expression reprogramming under short-term salt stress to investigate the shared and exclusive response patterns and expression connections of salt response genes in the roots of bermudagrass. Some key regulatory pathways, gene families and hub genes induced during the early stages of salt stress imposition were identified. These results could give an overview of the early-salt response transcription map and provide more useful information for further study of the salt response of bermudagrass.

## Results

### Effect of salt stress on the physiological parameters of bermudagrass roots

**Fig. 1:**
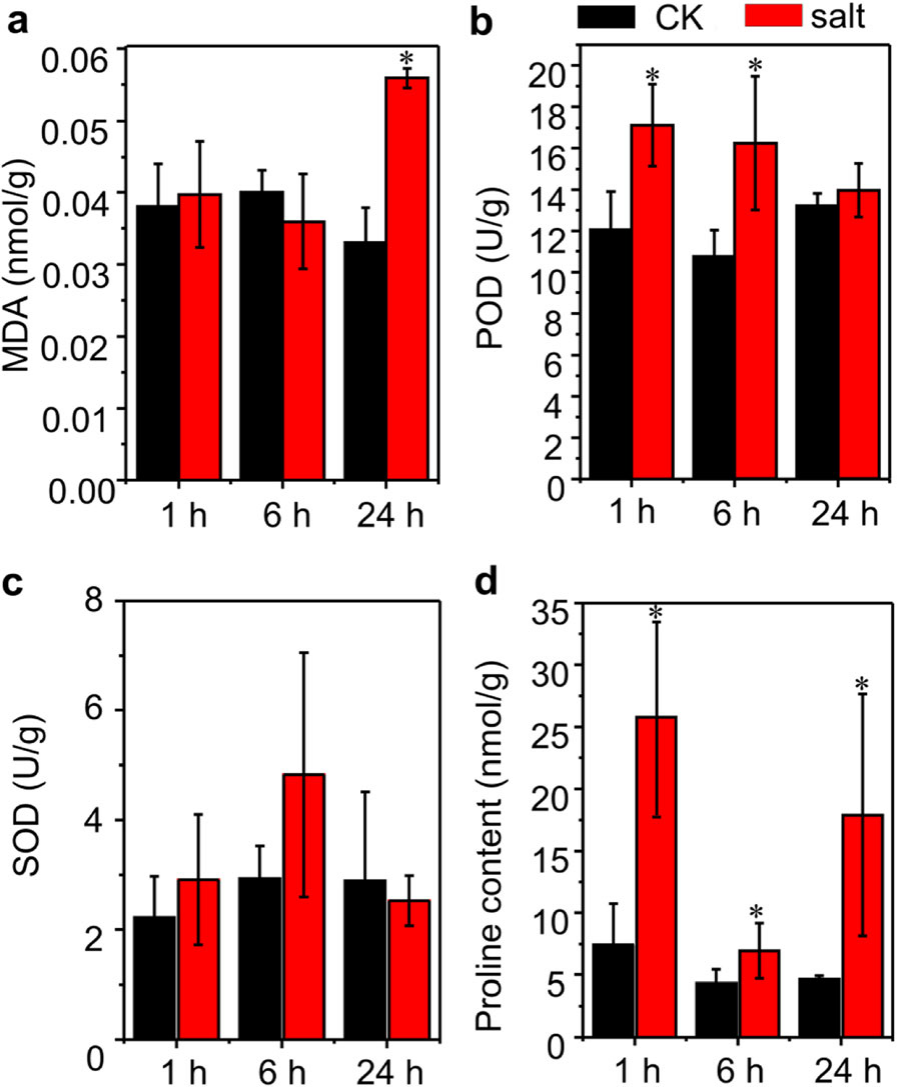
Physiological parameters of bermudagrass roots grown under control and salt conditions. The uniform stolons were planted in growth media for one month, and the plants mowed to the same height were then transferred into CK (0 mM NaCl) and salt stress (200 mM NaCl) conditions for 1 h, 6 h or 24 h in a hydroponic culture. The MDA content (**a**), POD activity (**b**), SOD activity (**c**) and proline content (**d**) were measured. Data are means ± *SD* of three independent experiments; * indicate statistically significant difference between control and salt stress under certain time point at *P* < 0.05 by Student’s *t*–test

To study the early salt response in the roots of bermudagrass, the plants were treated with 200 mM NaCl for 1 h, 6 h and 24 h respectively. The roots samples were collected for physiological parameters determination and transcriptome analysis. Due to a relatively shorter exposure time to salt stress, the growth parameters such as plant height, shoot biomass and root length were not significantly affected (data not shown). However, physiologically, the roots of 24 h salt-treated plants displayed higher malondialdehyde (MDA) content than control plants (Fig. 1a). The POD activity was significantly elevated in the roots of 1 h and 6 h salt-treated plants compared to that in their respective control regimes (Fig. 1b). The SOD activity of 1 h and 6 h salt-treated roots showed an upward trend, but the increase was not significant compared to their respective control plants (Fig. 1c). Besides, salt stress induced a higher proline accumulation of roots compared to non-salinity conditions (Fig. 1d). The accumulation of these metabolites indicated that the roots of plants were experiencing salt stress and producing a stress response at the time when used for transcriptome analysis.

### General transcriptomic responses and expression profiles of differential expression genes in the roots of bermudagrass

**Fig. 2:**
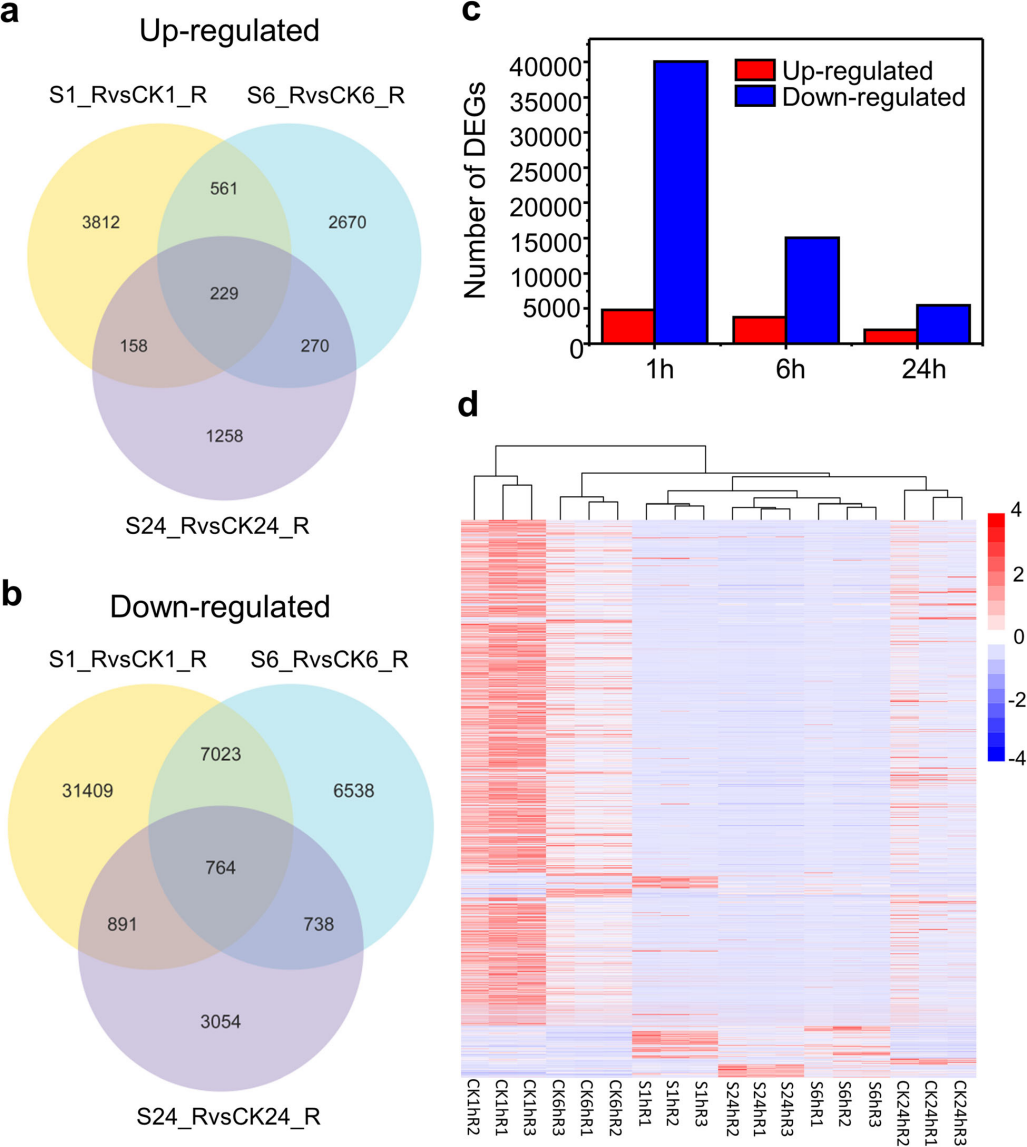
Summary of different expression genes after salt exposure at different time point. Venn diagram showing the overlap of up-regulated genes (**a**) and down-regulated genes (**b**) at various time points (1 h, 6 h, 24 h). The numbers of DEGs exclusively expressed in one sample are shown in each circle. The numbers of DEGs with a common tendency of expression changes between the two treatments are shown in the overlapping regions. (S1_R_salt, NaCl treated for 1 h; CK1_Control, without NaCl treated for 1 h; S6_R_salt, NaCl treated for 6 h; CK6_Control, without NaCl treated for 6 h; S24_R_salt, NaCl treated for 24 h; CK1_Control, without NaCl treated for 24 h). **c** The number DEGs under different time points. **d** A heatmap of the relative expression levels of of DEGs under different time point

Totally 695,542 transcripts and 694,799 unigenes with an N50 of 1391 bp were obtained. Gene expression analysis showed that the expression of 58,979 genes was significantly altered in response to salt stress at one or more time points. The Venn diagram indicated that 229 genes were up-regulated while 764 genes were down-regulated at all the three-time points (Fig. 2a, b). Among the up-regulated genes under salt stress, 3812, 2670, and 1258 genes were exclusively expressed in 1 h, 6 h and 24 h respectively (Fig. 2a). Among the down-regulated genes, the expression of 31,409 genes was regulated specifically at 1 h; the expression of 6538 genes was modulated only at 6 h whereas the expression of 3054 genes was exclusive for 24 h (Fig. 2b; Table S1). Overall, most of the responsive genes showed down-regulated expression by NaCl at all the three-time points, respectively (Fig. 2c). Also, the number of differentially expressed genes (DEGs) after 1 h salt treatment was relatively more than the number after 6 h and 24 h-salt treated (Fig. 2c, d).

**Fig. 3:**
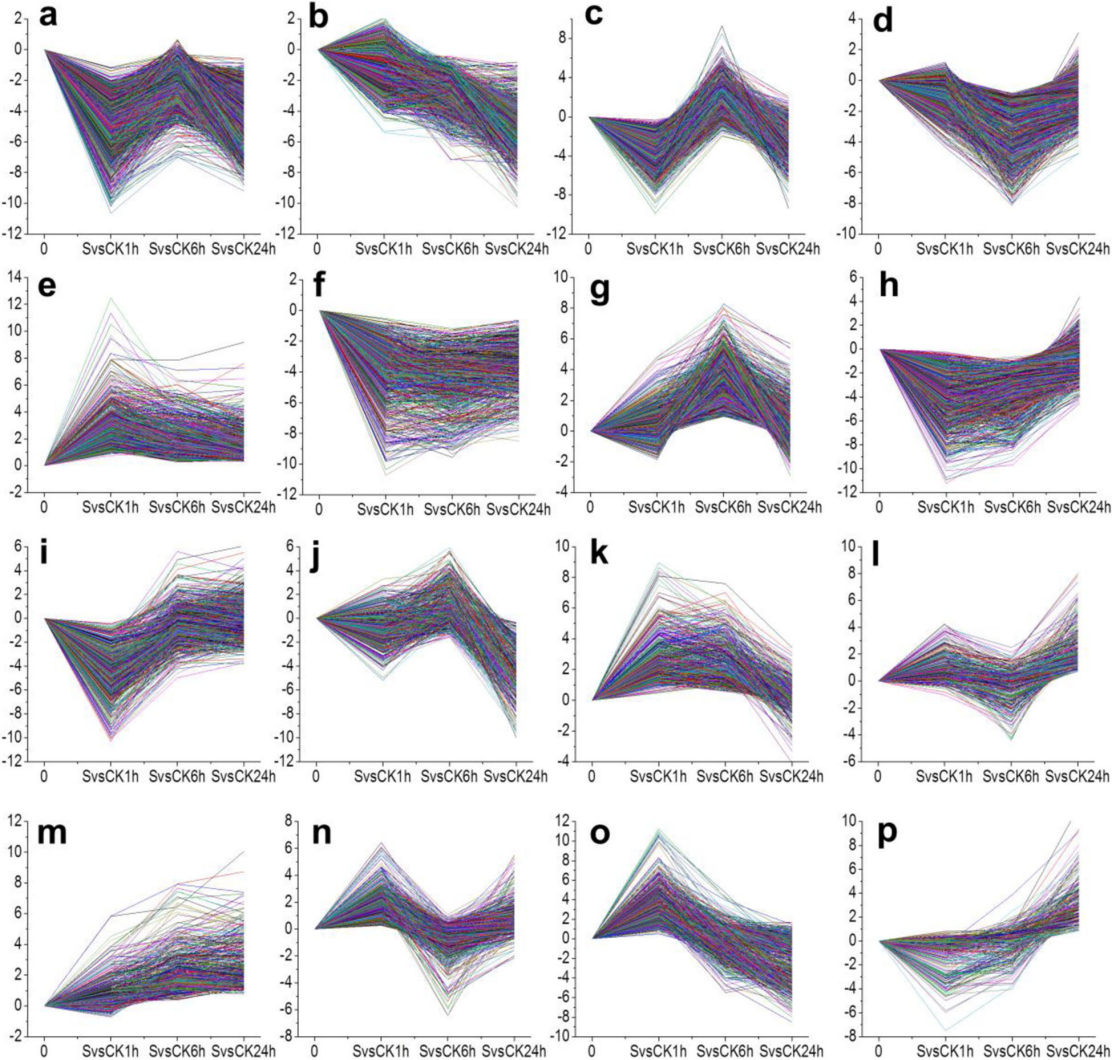
Different expression genes grouped according to temporal expression profiles using STEM software. DEGs were mainly divided into 16 distinct temporal profiles. Each of the profiles (**a-p**) is represented as a different plot, with ratios log_2_ Fold change (S vs CK) for each of the assigned transcripts at each time point

To detect the expression pattern of DEGs, the STEM (Short Time-series Expression Miner) software package (Table S1) was used and 16 distinct temporal expression patterns were identified (Fig. 3). The predominant profiles indicated that, following salt treatment, the expression pattern of the most DEGs changed rapidly within the first 1 h following salt treatment (Fig. 3). Some genes expression peaked (repression or induction) at 1 h (Fig. 3e, i, o) while other groups of genes peaked at 6 h (Fig. 3d, g) or 24 h respectively (Fig. 3b, j, l, m, p). Some genes responded at 1 h continued along the same trajectory at the subsequent time points (Fig. 3b, m) or reverted to untreated state levels (Fig. 3i, o). Some genes were induced at 1 h and persisted (Fig. 3e, f). Other genes displayed slight changes until 24 h (Fig. 3l, p). Also, as compared to 1 h or 24 h, some genes displayed converse patterns of induction and repression at 6 h (Fig. 3c, n). Moderate responses of some genes were observed at 1 h and 6 h and showed a slight response at 24 h (Fig. 3h, k). Still other genes repressed at 1 h were slightly repressed at 6 h and reached a highly suppressed expression at 24 h (Fig. 3a). These gene expression profiles indicated that there might be a time-specific response pattern in the roots of bermudagrass.

### Functional categorization of deferentially expressed genes

**Fig. 4:**
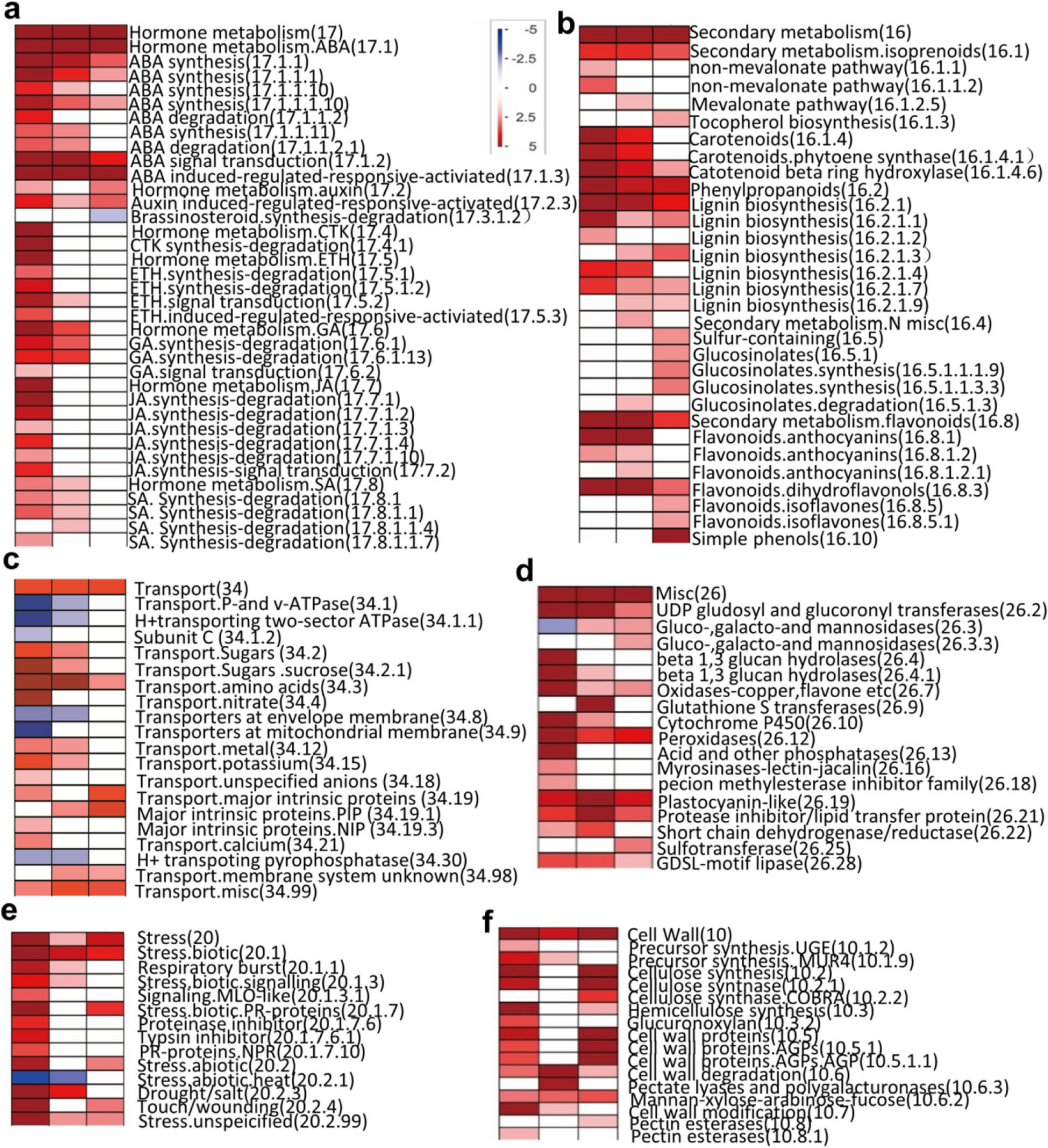
PageMan display of coordinated changes of selected gene categories activated by salt exposure. **a** Hormone metabolism, **b** Secondary metabolism, **c** Transport, **d** Misc, **e** Stress, **f** Cell wall. The log_2_ Fold change of SvsCK1h (left column), SvsCK6h (middle column) and SvsCK24h (right column) were subjected to over-representation analysis. Colour scale is: red, significant enrichment of up-regulated genes; blue, significant depletion of up-regulated genes. The complete analysis and its display are provided in Supplemental Fig. S6 and Table S2

**Fig. 5:**
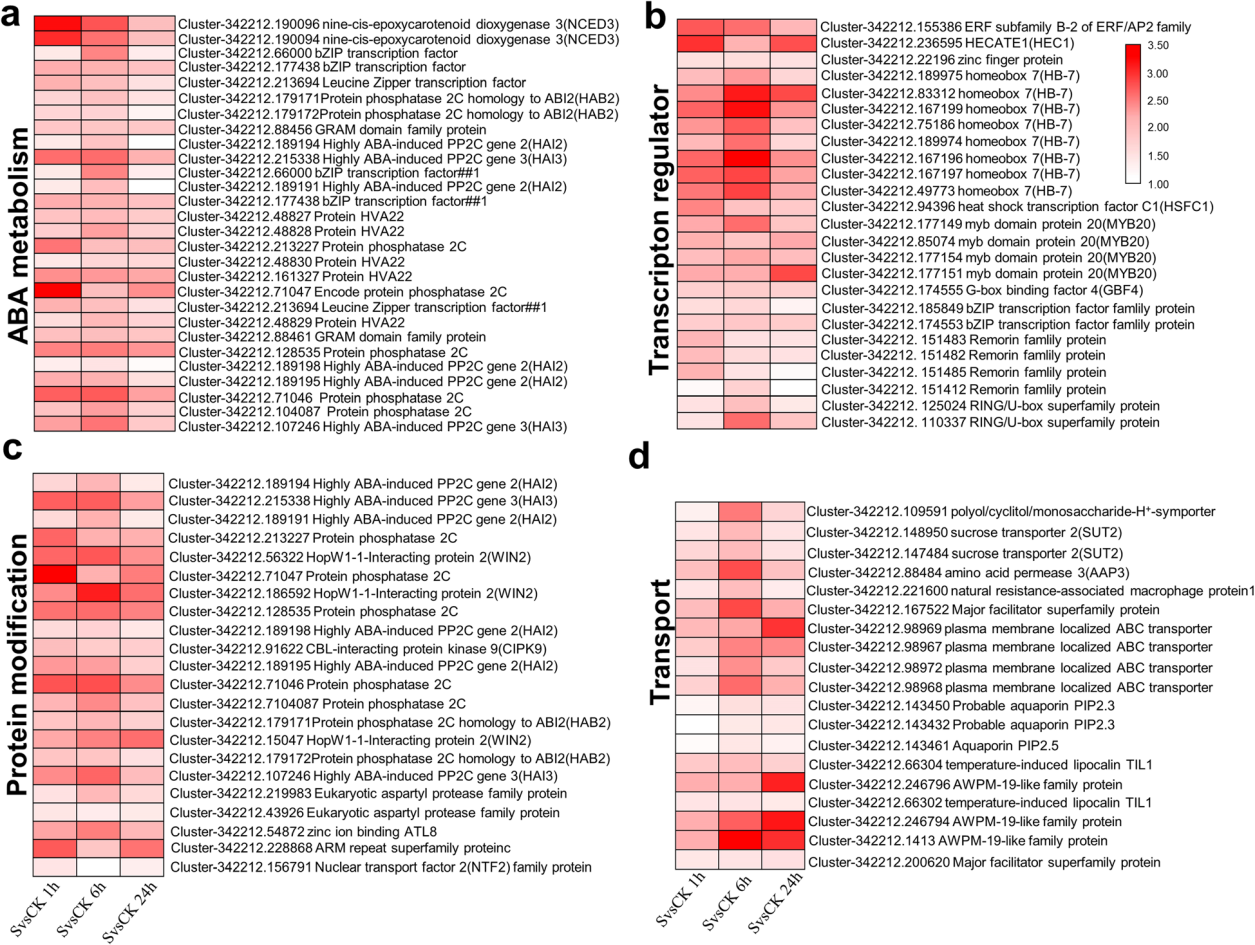
Expression of selected co-upregulated genes after salt stress displayed by Heatmap diagram. **a** Members of ABA metabolism-related gene. **b** Transcription regulators. **c** Protein modification-related genes. **d** Transporters. SvsCK1h (left column), SvsCK6h (middle column) and SvsCK24h (right column)

To understand the transcript pattern changes of genes belonging to different categories, and to detect the existence of differently expressed genes, PageMan analysis was then used to analyse the relationship between the enriched transcripts of different response time points and their biological significance. Results showed that bins involved in major metabolism (2), cell wall (10), secondary metabolism (16), hormone metabolism (17), stress (20), misc. (26), development (bin 33) and transport (34) were all up-enriched (Fig. 4a-f; Table S2) while the DNA (28), protein (29), energy-related (8: TCA/org transformation; 9: mitochondrial electron transport/ATP synthesis) and cell (31) related bins showed significant depletion of up-regulated genes under salt stress at all three time points (Fig. S1; Table S2). These consistently and continuously up-regulated gene categories mainly included genes that participated in ABA synthesis and signaling transduction (e.g. 9-cis-epoxycarotenoid dioxygenase, NCED; protein phosphatase 2C, PP2C; ABRE binding factors, ABFs) (Fig. 5a), transcription factors (e.g. members of HB, MYB and bZip) (Fig. 5b), several groups of transporters (Fig. 5d) (e.g. transporters of sugars, amino acids, peptides and oligopeptides; ABC transporters; multidrug resistance systems; major intrinsic proteins.PIP), genes regulating levels of osmoprotectants (e.g. S-adenosylmethionine decarboxylase; galactinol synthases; raffinose sythases; trehalose; callose; galactose), transcripts that encode antioxidant enzymes (e.g. peroxidase), genes involved in oxidases stress, such as oxidases-copper, glutathione S transferases, beta 1,3 glucan hydrolases, plastocyanin-like proteins) and other proteins (e.g. the late embryogenesis abundant proteins and AWPM-19-like membrane family proteins participated in osmotic stress response; genes involved in phenylpropanoids, carotenoids, flavonoids and polyamine metabolism such as 4-coumarate--CoA ligase 1 (4CL-like), peroxidase 1, phytoene synthase gene (*PSY3*) and 2-oxoglutarate (2OG) and Fe(II)-dependent oxygenase superfamily protein) (Fig. S2a; Table S1). Although the protein synthesis and amino acid activation sub bins showed significant depletion of up-regulated genes under all three time points, the protein modification sub-bin significantly enriched the up-regulated genes (e.g. members of PP2C, HAB, HAI, WIN, CIPK family) (Fig. 5c). However, genes involved in protein translational modification such as kinase and ubiquitination pathway-related genes were up-regulated (Fig. S3).

### Salt responsive genes categories at different time points

Salt treatment triggered exclusive response at different time points (Table S2). For instance, the receptor-like kinase sub-bin (30.2) involved in signaling bin (30) was specifically over-represented immediately after salt exposure for 1 h (Table S2; Fig. S4a), including receptors such as leucine-rich repeat (LRR V, VIII and XII), thaumatin-like, Catharanthus roseus-like RLK1, domain of unknown function (DUF) 26, legume-lectin domain (LLD), LRK10 like; lysine motif, proline extension-like (PERK), S-locus glycoprotein like and wall-associated receptor kinase (WAK). Some calcium signaling-related genes were specifically up-regulated immediately after the roots were exposed to salt for 1 h (e.g. calcium-dependent protein kinase, CDPK11; Calmodulin, CAM3; calmodulin-domain protein kinase, CPK5; calmodulin-like, CML43). A mitogen-activated protein kinase MAPK2 (cluster-342,212.26954), which is a homolog to At2g43790 was also up-regulated exclusively at 1 h (Fig.S2b). Sub bins involved in hormone metabolism (17) such as JA synthesis-degradation (17.7.1) and signal transduction (17.7.2), CTK metabolism (17.4) and ETH metabolism (17.5) were specifically induced at 1 h (Table S2; Fig. 4a). Genes that participated in ETH biosynthesis (one ACC synthase and four ACC oxidase), ethylene signal transduction (three ERF and one DREB), JA biosynthesis (one allene oxide synthase, AOS1; one allene oxide cyclase, AOC4), JA signal transduction (JAZ1) and CTK metabolism degradation (five UDP-glucosyl transferase and nine cytokinin oxidase) were significantly up-regulated under 1 h salt treatment, indicating that these hormones could be involved with early response to salt stress in the roots of bermudagrass (Table S2, Fig. 4a). In addition, a series of TFs sub bins (e.g. ARF: 27.3.4; ARR: 27.3.12; C3H: 27.3.5; NAC: 27.3.27; Trihelix: 27.3.30; AS2: 27.3.37; JUMONJI: 27.3.57; PHOR1: 27.3.64; Psudo ARR: 27.3.68) were over-represented after 1 h salt treatment compared to the later time points, implying that these TFs might be exclusively involved in early salt response in the roots of bermudagrass (Table S2; Fig. S5). Other genes were also exclusively expressed at 1 h, including several groups of transporters (e.G. *major* intrinsic proteins NIP, PIP); stress response molecules (e.g. typsin inhibitor, PR proteins, MLO-like receptors), lipid metabolism (e.g. choline kinase) (Table S2).

More map-bins enriched by PageMan were found exclusively over-represented after plants were exposed to salt for 1 h compared to the latter two-time points (16 at 1 h, 10 at 6 h and 11 at 24 h respectively) (Fig. S6). These included bins of tetrapyrrole synthesis (19) (Fig. S4c), biodegradation of xenobiotics (24) (Fig. S4e), lipid metabolism (11) (Fig. S4b), suggesting a relative earlier response to salt (Fig. S4). However, the polyamine synthesis sub-bin was over-represented only after 6 h and 24 h salt treatments (Fig. S6). In the secondary metabolism bin, the sub bins related to isoprenoid, phenylpropanoid and flavonoids metabolism were presented upregulated at all the three-time points of salt exposure. However, some sub-bins included in secondary metabolism (16) such as simple phenol (16.10), glucosinolates (16.5.1), isoflavones (16.8.5) and tocopherol biosynthesis (16.1.3) (Table S2) were exclusively over-represented at 24 h (Fig. 4b), indicating a slightly delayed response to salt. These results revealed that regulators or effectors involved in different salt-responsive categories might be active at different time point following the perception of salt stress.

### Coexpression network analysis and hub gene investigating by WGCNA

**Fig. 6:**
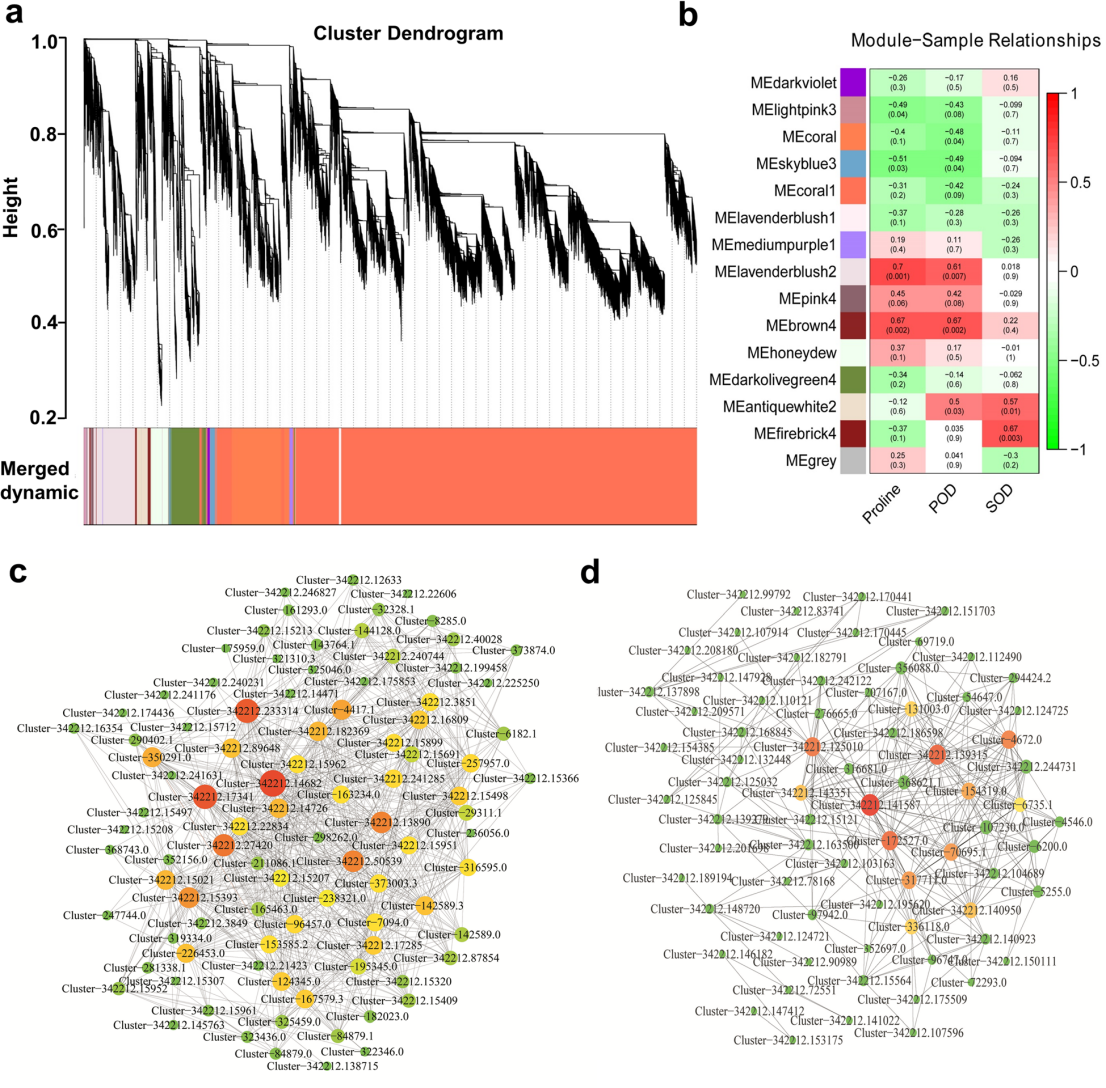
Weighted gene co-expression network analysis of salt-related physiological indicators associated genes. **a** Fifteen modules of co-expressed genes showed on Hierarchical cluster tree. A leaf represents each of the DEGs and a major tree branch represents each of the fifteen modules. Modules in designated colours. The modules in designated colours were shown in lower panel. **b** Correlation between gene expression modules and physiological parameters (proline, POD and SOD) in the whole co-expression gene network. The left panel shows fifteen modules. The correlation value and the *p* value were shown within each cell. The right panel is a colour scale for module trait correlation from − 1 to 1. Visualization of key co-expression network lavenderblush2 module (**c**) and brown4 (**d**) module by Cytoscape. Key hub genes identified by WGCNA indicated by larger and red circles

WGCNA was further performed to identify the specific genes that are highly associated with salt response in the roots of bermudagrass (Table. S4). Based on pairwise correlations analysis of gene expression, fifteen network modules in the co-expression network, designated darkviolet, lightpink3, coral, skyblue3, coral1, lavenderblush1, mediumpurple1, lavenderblush2, pink4, brown4, honeydew, darkolivegreen, antiquewhite2, firebrick4 and grey were identified (Fig. 6a, b). Investigating the relationships between salt response physiological indexes (proline\POD\SOD) and module eigengenes revealed that the correlation coefficients value varied from − 0.67 to 0.70 in proline, from − 0.55 to 0.70 in POD and from − 0.55 to 0.70 in SOD (Fig. 6b). At the *p* value < 0.05 level, four modules were associated with proline content, while five modules with POD and two modules with SOD. The eigengenes of lavenderblush2 and brown4 modules showed significant positive correlations (*p* < 0.01) with proline and POD, suggesting these two modules might have greater relevance in salt response (Fig. 6b). Further, the lavenderblush2 and brown4 modules, representing 882 and 438 genes respectively, were visualized with the Cytoscape software. The top three hub genes of brown4 co-expression network contained one homologue of hypothetical protein MTR_3g035650 from *Medicago truncatula* (Cluster-342,212.125010), one hypothetical mitochondrion protein homolog to AGC78945.1 from *Vicia faba* (Cluster-342,212.139315) and one classical transcription factor HSF (cluster-342,212.125010) (Fig. 6c; Table S4). The top three hub genes of lavenderblush2 co-expression network visualized by Cytoscape contained one gene encoding β-amylase (Cluster-342,212.182369) which belong to glycosyl hydrolase family 14 (Fig. 6d; Table S4). However, the other hub genes of these two modules were not annotated.

### RT-qPCR validation of selected deferentially expressed hub genes

**Fig. 7:**
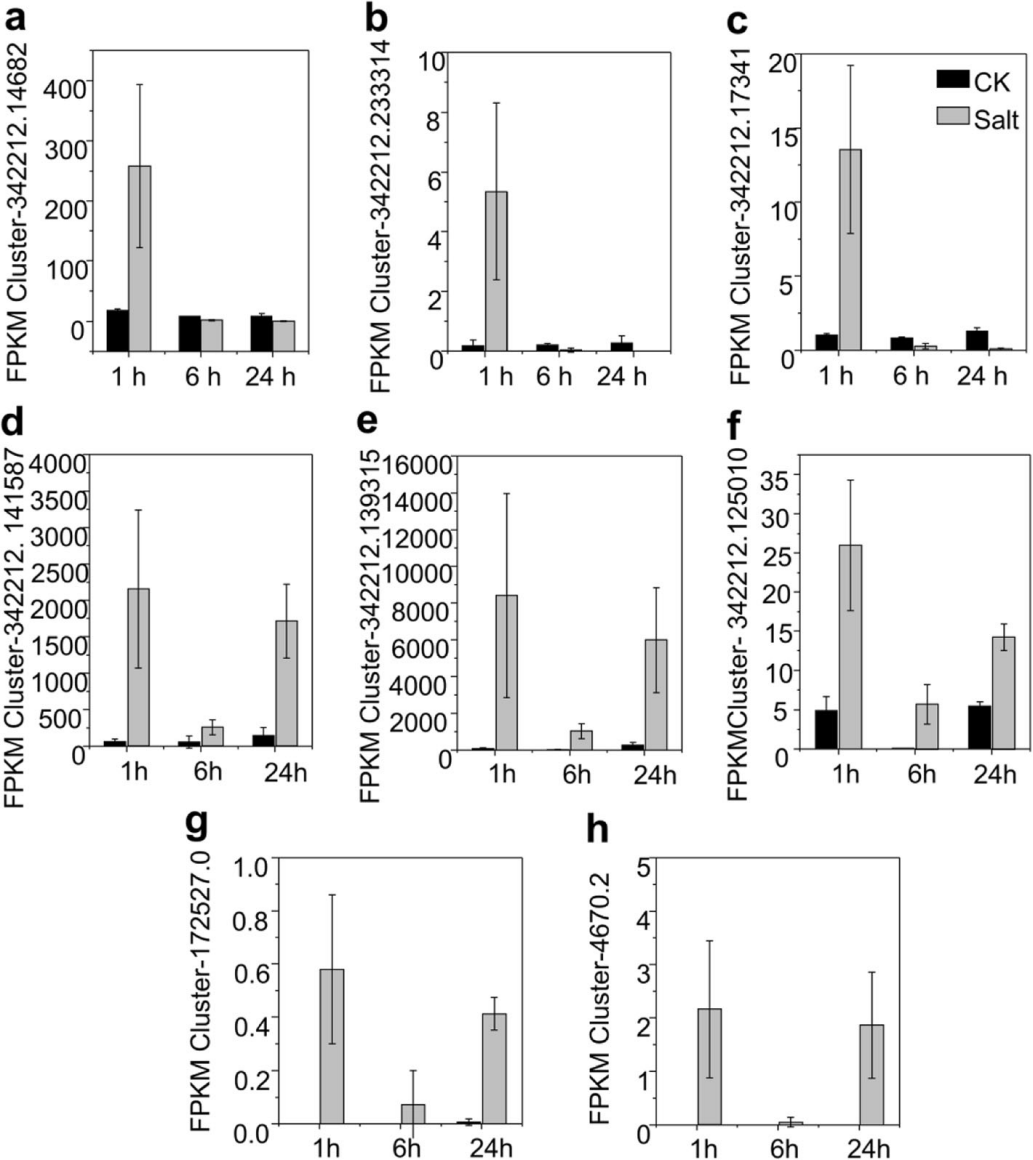
Transcript abundance of hub genes in the lavenderblush2 and brown4 modules. **a-c**, Hub genes from lavenderblush2 module. **d-h**, Hub genes from brown4 module. Transcript abundance is presented as the mean ± SE of FPKM values, *n* = 3 at each time point

Because hub genes were investigated based on the relationship between FPKM of genes and the physiological parameters, the accuracy of FPKM value of these hub genes in the transcriptome data should be confirmed. The expression levels of eight hub genes with different expression pattern (Fig. 7) from two WGCNA modules lavenderblush2 and brown4 were further determined by RT-qPCR analysis (Fig. S7; Table. S5). The expression profiles of top three hub genes from the module lavenderblush2 showed an induced expression after 1 h salt stress but a decreased expression or no obvious alteration at the latter time points (Fig. 7a-c; Fig. S7a-c). The expression of top five hub genes from the module brown4 showed a significant induction at all the three-time points under salt exposure (Fig. 7d-h; S7d-h). The expression patterns of these selected hub genes verified by RT-qPCR and their FPKM values from the transcriptome showed a consistent trend under the corresponding treatments. These results not only confirmed the differential response pattern of these hub genes from different modules but also confirmed the reliability of the transcriptome data.

## Discussion

### Time-specific quick salt response modules in the roots of bermudagrass

Previous transcriptome analysis of plants reveals differential response strategy at different stages of salt stress [31, 32]. For instance, plants response to the initial osmotic stress by increasing the intracellular concentrations of osmolytes [2]. After NaCl exposure for 24 to 72 h, alleviating Na^+^ toxicity raises to a more urgent task [23, 24]. To investigate the transcriptome adjustments of bermudagrass roots to the salt shock in the early phase, 1 h was firstly chosen to study the immediate salt response. We next chose 6 h as a treat time point to investigate the immediate following reaction after the earliest response to salt (1 h) based on the previous study showing that soybean faced to an initial osmotic stress stage in 1 h to 4 h after salt treatment [2]. Moreover, 24 h was still chosen to investigate if the salt response strategy begins to change in bermudagrass because 24 h might be a turning point at which the salt response strategy might begin to change in some plants [23, 24].

In bermudagrass, about 2.4 and 6 times more specific salt-responsive genes derived from different gene categories were differentially regulated in the roots exposed to salt for 1 h compared to those exposed to salt for 6 h or 24 h respectively, suggesting a quick response after salt exposure (Fig. 3). For example, several signal receptors like kinases (e.g. LRR, thaumatin-like, RLK1, DUF26, LLD, LRK10 like, PERK, and WAK) were detected immediately and exclusively up-regulated at 1 h (Fig. S4a). These signal receptors kinases always response at an earlier time point to function in protein phosphorylation and modification, which is an important step in initiating salt response signalling pathways and ultimately leading to a transcriptional regulation [33–37]. Moreover, the salt signal could also immediately trigger the downstream hormones pathways, which are known to be involved in stress responses in a wide range [19, 38]. In this study, genes involved with ABA biosynthesis and signal transduction sub-bins (17.1.1, 17.1.2, 17.1.3) were consistently up-enriched at all three-time points (e.g. NCED; PP2C and ABFs) (Fig. 5a), suggesting the established role to salt response [9]. However, we also noticed that transcripts involved in the metabolism of biosynthesis and signal transduction of ETH (e.g. ACC synthase; ACC oxidase and ERF) and JA (e.g. AOS1 and AOC4), were exclusively over-represented at 1 h of salt exposure (Fig. 4a; Table S2), indicating that these salt responsive hormones metabolism pathways might participate in the quick salt response progress in the roots of bermudagrass [32, 39, 40]. In addition, the induction of transcripts involved in CTK and GA degradation was noticed (Fig. 4a; Table S2). Transcripts encoding gibberellin-degrading enzyme gibberellin 2-oxidase (homologs of At4g21200 and At1g75450 respectively) suggested the cell growth were partly inhibited to survive under environmental salt stress. The expression of at least 9 transcripts of AtCKX6 (At1g75450) homologs were regulated (Table S2), which encoding a cytokinin oxidase/dehydrogenase that participates in catalysing the degradation of cytokines [41, 42]. These results suggested that hormone signaling does not work alone while mediating salt response but might function in multifarious crosstalk network with other hormones.

Intracellular phosphorylation events are downstream of secondary messengers, such as CDPKs [9–14] and MAPK cascades [43–45], which are reported to be essential sensor-transducers in plants. In this study, some gene members involved in calcium signaling responded immediately after salt exposure for 1 h (e.g. CDPK11, CAM3, CPK5 and CML43) (Fig. S4a; Table S2). Some calcium-transporting ATPase encoding genes were specifically over-represented at 1 h, which could further promote the transmembrane transport of Ca^2+^ (Table S2). A *MAPK2* gene (cluster-342,212.26954), which is a homolog to At2g43790 was also up-regulated exclusively at 1 h (Fig. S2b) and might interplay with ROS and hormone in salt response [46, 47]. The immediate up-regulation of these protein kinases encoding genes might further trigger downstream transcriptome reconfiguration to cope with the stressful salt condition [48].

In the roots of bermudagrass, we also identified over ten transcription factor families, which were significantly induced at one or more time points after salt exposure (Fig. S5). The induced TFs number was much more at 1 h than latter time points. Among those TFs, AP2, WRKY, bHLH and HB families accounted for a large ratio of the total number of salt-induced TFs identified and the three families (MYB, HB, bZip) were significantly induced at all three time points (Fig. 5b; Table S2). One HSF transcription factor was investigated as a hub gene of brown4 co-expression network in this study (Fig. 6d). The expression of this HSF transcription factor showed up-regulated by salt at all three time points and it could be a good target for future studies (Fig. 7f; S7). Consistent with the previous studies that WRKY TFs could positively or negatively participate in salt tolerance [49], we also observed that 20 of the 23 WRKY TFs detected significantly induced in response to 1 h salt treatment in the roots (Fig. S5; Table S2). The AP2/EREBP family was also reported to include some stress-responsive TFs [50]. We also observed that 16 of 17 AP2 transcripts were up-regulated after 1 h salt treatment (Table S2). Under salt stress, another most affected TF family in the roots was bHLH, with 24 of 28 transcripts being induced at 1 h and 10 of 19 were increased at 6 h by salt stress (Table S2). Among these induced bHLH TFs, some important members which have been reported to positively participate in salt stress response such as bHLH92 [51]. The Aux/IAA families were significantly enriched in salt-responsive transcripts especially at 1 h with all 12 transcripts all up-regulated by salt stress (e.g. IAA5, 12, 20, 24, 18, 23) (Table S2). These salt response Aux/IAA genes have a central role in auxin response and might act to integrate the signal from environmental stimuli into the auxin-related gene regulatory network [52]. Therefore, here, we noticed that some biological processes responded at the early stage of salt stress, mainly including signal transduction, hormone metabolism and regulation of TFs. These quick responses might then form a cascade to active a series of downstream response factors.

### Common and distinctive positive salt response mechanisms in the roots of bermudagrass

Plants have evolved diverse gene families to detoxify ROS caused by harsh environments such as salt [19, 20]. In our study, the POD activity was significantly higher in the roots of 1 h and 6 h salt-treated plants compared to that in their respective control roots (Fig. 1b). However, the SOD activity of 1 h and 6 h salt-treated roots showed an upward trend, but the increase was not significant compared to their respective control plants (Fig. 1c). Accordingly, a few members of POD encoding genes were up-regulated but SOD encoding genes were not up-regulated in our transcriptome data (Fig. 4d; Table S2). Because oxidative stress is a consequence of the deterioration of lipid peroxidation (indicated by MDA) brought about by ROS, we also measured the MDA content in the roots. However, the roots MDA content displayed a higher value than control plants until exposed to salt for 24 h (Fig. 1a), suggesting a progressive accumulation with the increased treatment time. Other members of gene families encoding oxidases-copper, glutathione S transferases, beta 1,3 glucan hydrolases, UDP glucosyl and glucoronyl transferases, plastocyanin-like proteins (Fig. 4d; Table S2) were also up-regulated at one or more time points to cope with the salt stress. For example, UDP glucosyl transferases UGT79B2/B3 in *Arabidopsis* was reported to contribute abiotic stress tolerance such as salt and drought via affecting anthocyanin accumulation and enhancing ROS scavenging [53]. Consistent with the previous studies in plants, some bioactive secondary metabolites in the roots of bermudagrass (e.g. carotenoids, tocopherols and flavonoids) [54–56] were also over-represented under salt and might also serve as ROS scavengers (Fig. 4b; Table S2). As expected, the genes regulating osmoprotectants levels were also highly upregulated in this study. They included genes encoding galactinol synthases, raffinose synthase, trehalose, callose and galactose (Fig. S4d), which were reported to be the first stress-inducible genes under salt stress [23–26].

The plant cell wall consists of cellulose, hemicellulose, lignin, pectin and many glycoproteins [57, 58] and is considered to be an important factor to sense and response to salt stress. We also noticed that genes involved in cellulose synthase (10.2), hemicellulose synthesis (10.3) and lignin synthesis (16.2.1) were over-represented in the salt-treated roots of bermudagrass (Fig. 4f). The expression of glycoside hydrolase (GH17) family genes was significantly induced under 1 h of salt stress (Fig. 4d; Table S2), suggesting it may participate in the post-translational modifications of cell wall-related proteins and lead to the alteration of cell wall flexibility [59, 60]. In addition, other cell-wall related gene families which function in cell wall extensibility were also showed differential regulation in salt responsive transcripts. For example, the expression of *MUR4* was found up-regulated in the roots of bermudagrass (Fig. 4f), and was reported to function in the biosynthesis of UDP-arabinose. Mutation in *MUR4* affects cell wall integrity and leads to an defective cell-cell adhesion with a reduced root elongation under high salinity [61]. Moreover, several AGPs (arabinogalactan proteins) encoding genes were found up-regulated by salt at the transcript level in our study (Fig. 4f). The AGPs on cell walls or plasma membranes are also reported to be associated with cell growth [62, 63] and one AGP (SOS5) was known to contribute to salt tolerance in *Arabdiopsis* [64]. We further noticed an earlier response of lipid metabolism in the roots of bermudagrass. In particular, the expression of genes involved in FA synthesis and elongation were down-regulated while genes involved in FA desaturation and lipid degradation were significantly up-regulated immediately when exposed to salt for 1 h (Fig. S4b). Studies have shown that FA desaturases play an important role in the maintenance of the biological function of membranes in plant cells under different conditions including salt stress [65, 66]. Here, salt stress markedly changed the expression of genes encoding ω-3 FA desaturases which might lead to an alteration of FA composition (Fig. S4b, Table S2). The immediate regulation of genes coding for a recombination of lipid composition can provide novel insights to improve salt tolerance of bermudagrass.

Other than secondary metabolisms-related genes which significantly participated in cell wall modification (Fig. 4f), some important secondary metabolism pathways were significantly induced at a prolonged time point, suggesting slightly slower reactions that may involve metabolic adjustment [67, 68]. For example, the polyamine synthesis sub-bin was over-represented only after 6 h and 24 h salt treatments. Some sub-bins included in secondary metabolism such as simple phenol, glucosinolates, isoflavones and tocopherol biosynthesis were specifically over-represented at 24 h (Fig. 4b; Table S2). These secondary metabolisms were previously reported to be involved in plants oxidative response in some species [67, 68]. For example, the expression of laccase encoding genes was found up-regulated especially when exposed to salt for 24 h, which might participate in the oxidation and reduction of simple phenols in the roots of bermudagrass and alleviate the oxidize stress caused by salt stress [69, 70].

### Categories of down-regulated genes by salt stress in the roots of bermudagrass

In this study, down-regulated genes were more abundant at all three time points respectively (Fig. 2c), suggesting an impact of the huge negative regulation of transcription on plant metabolism and functioning. Actually, important enriched gene categories such as hormone metabolism, transcription factors, misc. and secondary metabolism also contained large number of down-regulated genes (Table S2). For instance, genes involved in brassinosteroid synthesis or degradation (e.g. CYP450 family members) and signal transduction (e.g. BRI) were significantly down-regulated by salt stress (Table S2), suggesting an interaction of hormones to participate in salt response in bermudagrass [71]. Although a series of TF families showed up-regulation, other TF families such as C_2_H_2_ and HAP showed a large number down-regulated genes at one or more time points (Table S2). HAP transcription factor AtHAP3b and C_2_H_2_ protein Zat7 were previously reported to play key roles in primary root elongation to promote drought tolerance and in salt resistance in *Arabidopsis,* respectively [72, 73].

In previous proteomic studies, NaCl treatment decreased protein translation, which is consistent with the downregulation of most ribosomal proteins related transcripts in this study (Fig. S3; Table S2) [27, 74]. We also noticed that the number of DEGs after 1 h salt treatment was relatively higher than the number after 6 h and 24 h salt treated (Fig. 2c, d). More ribosomal proteins encoding genes and protein metabolism related genes also were significant over-represented in down-regulated genes immediately after exposed to salt for 1 h, suggesting that more genes involved in protein or amino acid metabolism were quickly and negatively regulated. Genes involved in protein translational modification such as kinase and ubiquitination pathways were up-regulated (Fig. S3). Notably, the majority of E3 RING and E3 SCF proteins related genes were significantly induced by salt stress (Fig. S3; Table S2), suggesting that these enzymes may function in ways that might be independent on the 26S proteasome during salt response [75]. The inhibited protein synthesis and enhanced protein degradation might hike the concentration of free amino acid, especially proline, which can act as an osmotic protective substance. In this study, the proline content in the roots of bermudagrass was significantly induced after NaCl exposure (Fig. 1d). These free amino acids could further initiate synthesis of dehydrin or polyamine, which might function in the maintenance of the structure of the protein and cell membrane under salt [2]. However, proline synthetic related category was not significantly over-represented, suggesting that genes involved in proline metabolism might not receive significant transcriptional regulation at all treat time points in this study.

**Fig. 8:**
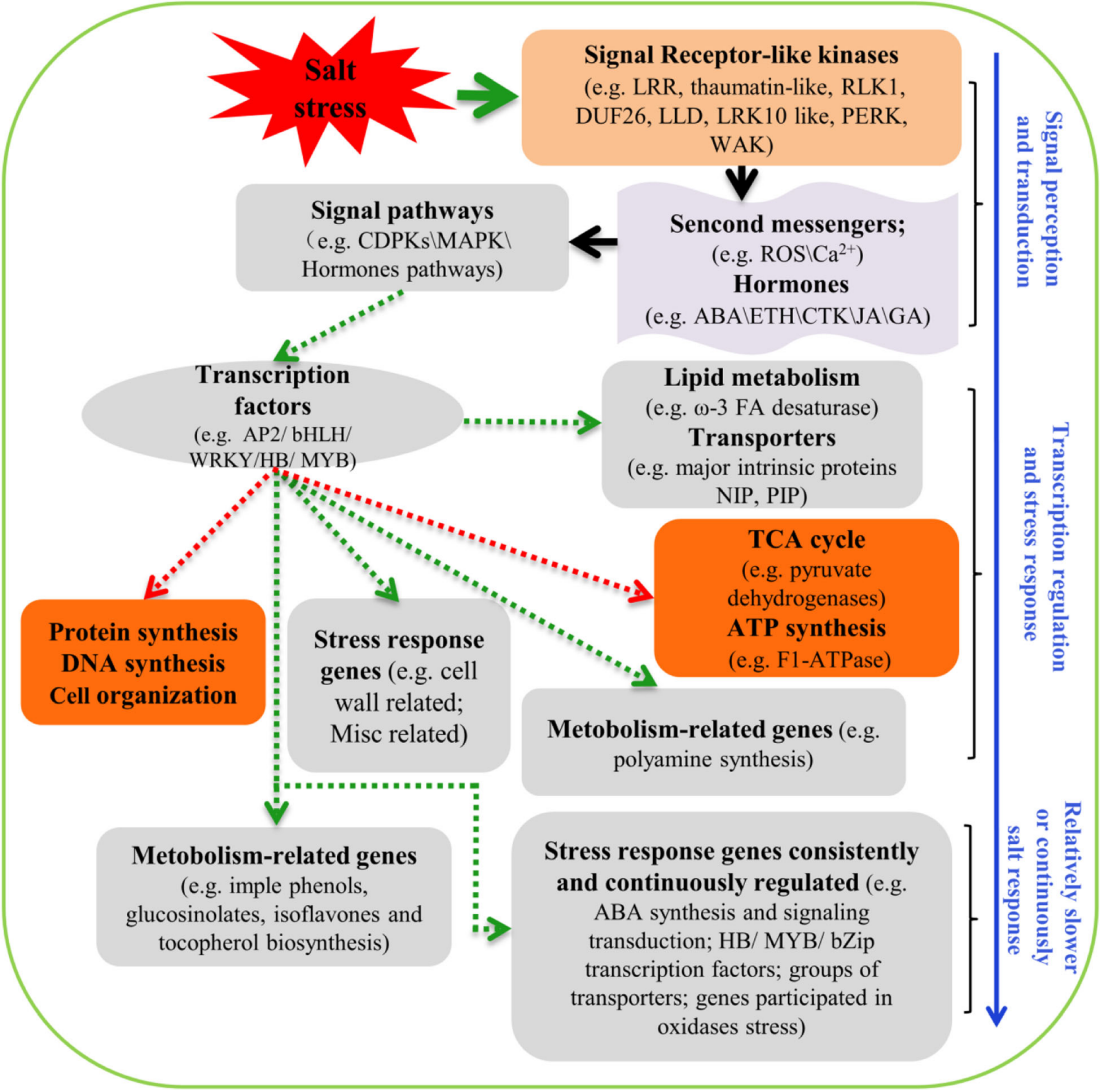
A proposed model of key gene categories significantly affected by salt in the roots of bermudagrass. Red arrows indicated the main negatively affected gene categories after salt stress and green arrows indicated some positively regulated gene categories. The blue arrow indicated the time course of salt response from signal perception and transduction to later salt response

Moreover, salt stress downregulated the expression of genes involved in tricarboxylic acid cycle (TCA), which is the main respiratory pathway in aerobe organisms (Fig. S1a; Table S2). For example, genes encoding pyruvate dehydrogenases, which function in the conversion of pyruvate to acetyl-CoA and thereby links the glycolytic pathway to the TCA cycle, were enriched among down-regulated gene categories (Fig. S1a; Table S2). Also, genes encoding the mitochondrial electron transport chain components such as NAD(P)H dehydrogenases and F1-ATPase were also exclusively enriched among down-regulated gene categories (Fig. S1b; Table S2). This suggests that the mitochondria might be damaged by oxidative stress. Also, we noticed that genes involved in DNA synthesis and cell organization were down-regulated especially at 1 h and 6 h (Fig. S1c, 1d). These gene categories might function together to save energies and materials to maintain plants growth and development under salt stress. A proposed model of key categories of genes positively and negatively affected by salt stress in the roots of bermudagrass was provided (Fig. 8). Generally, genes belonging to signaling pathways, involved in signal perception and transduction, such as signaling receptor kinase, hormone, and signal pathways, respond immediately after NaCl exposure. The transcription factors that respond at an earlier time point further positively or negatively regulate the downstream response genes. In these salt-responsive gene categories, some categories such as lipid metabolism and protein synthesis respond much earlier while other categories involved in secondary metabolite biosynthesis respond at latter time point [26, 76].

## Conclusions

Here, to understand the underlying regulatory mechanism following salt exposure in bermudagrass roots, a comprehensive transcriptome analysis was conducted. Groups of important salt response gene categories were identified. In addition, the distinctive salt-response pathways, time-specific response and potential hub genes investigated in this study can provide useful references to further study the salt response mechanism of bermudagrass in depth.

## Methods

### Plant materials and growth conditions

Bermudagrass accession ‘A12359’ (provided by Dr. Yanqi Wu in Oklahoma State University) was used in this study. Uniform stolons (ten stolons per pot) were planted in pots filled with sand for about 1 month. The plants were irrigated by Hoagland’s solution every 2 days. The roots of the plants were washed clean and transferred into a hydroponic culture with Hoagland’s solution for about 1 week to make the plants to adapt. Before treatments, the plants were mowed to the uniform height and transferred into a hydroponic culture consisting of CK (Hogland solution with 0 mM NaCl) and salt stress (Hogland solution with 200 mM NaCl) conditions. The plants were then treated for 1 h, 6 h and 24 h respectively. Each treatment comprised three replications and 18 root samples were collected for RNA extraction and physiological parameters measurement. The hydroponic culture was processed under the following conditions: 22/18 °C (day/night), 65% relative humidity, 16 h photoperiod with a photon flux density of 300 μmol m^− 2^ s^− 1^.

### Transcriptome analysis

Root samples of 1 h, 6 h and 24 h NaCl treated plants (Salt 1 h_R; Salt 6 h_R; Salt 24h_ R) and their respective control regimes (CK 1 h_R; CK 6 h_R; CK 24 h_ R) were used for transcriptome analysis. Each treatment comprised three replications and 18 sequencing libraries were prepared for RNA sequencing. Total RNA extraction was conducted following the Spectrum Plant RNA extraction kit (Sigma-Aldrich, USA). The RNA concentration was checked after DNase I digestion (NanoDrop ND-1000 UV-Vis spectrophotometer). The RNA integrity was measured using Bioanalyzer 2100 system (Agilent Technologies). The following steps were conducted as described before [31]. Generally, RNA libraries were generated according to NEBNext® Ultra™ RNA Library Prep Kit for Illumina® (NEB, USA). RNA fragments were attached to sequencing adaptors and sequenced on an Illumina HiSeq 2000 platform (Illumina, USA) to generate paired-end reads. Clean reads were generated after removing raw reads and adapters of all samples and then were de novo assembled by Trinity program to get assembly transcriptome [77]. The cufflinks program (version 2.0.2) was used to analysis the expression of transcripts and FPKM value were used to estimate their expression distribution [31]. DEGs (defined as genes up- or down-regulated by salt at one or more time points) were identified by comparing the expression alterations of the control and NaCl-treated samples. The significance of differential gene expression was assessed according to the following thresholds after salt treatment: log_2_ fold change value ≥1or ≤ − 1 and FDR (false discovery rate) ≤0.05 and FPKM value ≥1 [31].

### PageMan analysis

The log_2_ Fold change of SvsCK1h (left column), SvsCK6h (middle column) and SvsCK24h (right column) were imported into PageMan (a new version included in MapMan which was used for pathway analysis) and subjected to a comparative overview of over-representation in all of the treatments [78, 79]. To predict BINs significantly affected, we applied the statistical analysis provided in PageMan. The data was analyzed by Wilcoxon test. Significant differences of BINs were defined based on a *p*-value < 0.05. Red colour indicates a significant enrichement of up-regulated genes and blue colour indicates a significant depletion of up-regulated genes.

### Gene expression pattern analysis by STEM

For temporal expression profiles analysis, STEM software was used [80]. The DEGs whose salt-treated (S): untreated control (CK) log_2_ expression ratio differed significantly from 0 at one or more time points were used for analysis. The log_2_ expression ratio of genes was listed in Table S1. The maximum number of profiles was set to 16 and maximum unit change was set to 3 according to the method described before [27].

### WGCNA

WGCNA analyses were conducted using the expression of genes against the physiological parameters from the same samples by hypergeometric tests [81]. Generally, the RPKM values were firstly normalized by square root transformation and the cutoff for significant enrichment was FDR < 0.05 [82]. Network construction and module detection were conducted using the automatic one-step method with default settings. Then, the calculated module eigengene value was used to determine the association of modules with each salt responsive-related physiological parameter of 18 samples. Using Cytoscape, the modules which showed greater relevance with physiological parameters were visualized. The genes connecting to a greater number of genes was denoted with bigger size and dark red and were speculated to be more important for their interaction with other genes.

### Analysis of gene expression by RT-qPCR

The total RNA was extracted from the roots of three biological replicates at all three time points using RNeasy kit (Qiagen) according to the manufacturer’s instructions. First-strand cDNA of each sample was synthesized from DNase I-treated total RNA (1-5 μg) using TaqMan reverse transcription kit (Applied Biosystems). Each RT-qPCR reaction in a total volume of 20 μl contained 2 μl of cDNA template, 0.2 μM of primers and 10 μl SYBR Green qPCR mix (Toyobo, Japan) and was conducted using ABI real-time PCR system (Applied Biosystems, FosterCity, CA) as described before [30]. Each reaction had three technical duplications. Transcript pattern of each gene was determined by following the 2^-ΔCt^ method [83]. The *CdActin2* was used as a reference gene for gene expression normalization. All the technical aspects of RT-qPCR experiments fitted the requirements of MIQE Guidelines [84]. Before gene expression analysis, RNA integrity of all samples was evaluated on agarose gels electrophoresis and RNA absorbance OD260/280 ratios were examined. The original cDNA of each sample was made tenfold serial dilutions (10, 100, 1000x) with sterile ddH_2_O. The original cDNA and diluted cDNA were used as template to make standard curves (Plots of log gene copy number versus Ct value) to further calculate the gene-specific primer efficiency and R2 (regression coefficient) [85]. The primer efficiency of all primers used in study was over 90%. The specificity of each primer was confirmed by the single peak of the melting curve of all samples. The primers used were listed in Table S5.

### Statistical analysis

One-way ANOVA was performed using SPSS17.0 for Windows (SPSS). All of the above tests had at least three independent replicates. Results were expressed as mean ± *SD*, and * show significant differences *(P* < 0.05) by Student’s *t*–test.

## Supplementary Information

**Additional file 1: Fig. S1**. Example of down-regulated gene categories following salt treatment at different time points using PageMan. **Fig. S2**. Examples of genes commonly up-regulated at three-time points or specifically up-regulated at 1 h. Gene IDs and are indicated at the right side of each heat map. **Fig. S3**. Protein metabolism regulated following salt treatment at different time points using PageMan. SvsCK1h (left column), SvsCK6h (middle column) and SvsCK24h (right column). **Fig. S4**. Example of gene categories specifically up-regulated following 1 h salt treatment using PageMan. SvsCK1h (left column), SvsCK6h (middle column) and SvsCK24h (right column). **Fig. S5**. Transcription factors up-regulated following salt treatment at different time points using PageMan. **Fig. S6**. The complete view of enriched gene categories using pageman analysis. SvsCK1h (left column), SvsCK6h (middle column) and SvsCK24h (right column). **Fig. S7**. Relative expression levels of hub genes in the lavenderblush2 and brown4 modules. a-c, Hub genes from lavenderblush2 module. d-h, Hub genes from brown4 module. Relative expression level is presented as the mean ± *SD* of three replicates at each time point. Expression levels of hub genes were calculated using the 2^−△CT^ approach using *Actin2* as the reference gene. **Table S1**. The list of DEGs genes under different time point respectively and the fold change of DEGs for STEM package. **Table S2**. The list of time-specific enriched sub-bins of DEGs using MapMan systems. The genes from different enriched categories at each time point are given in log_2_ scale, and can be distinguished by the column headers. **Table S3**. The list of DEGs commonly regulated at all three time points. The genes from different enriched categories at each time point is given in log_2_ scale. **Table S4**. The physiological index used for WGCNA analysis and hub genes involved in lavenderblush2 and brown4 modules. **Table S5**. The primes of hub genes used for RT-qPCR.

## Data Availability

All data generated or analyzed during this study are included in the article with its supplementary material. We have deposited our transcriptome data in Sequence Read Archive (SRA) (http://www.ncbi.nlm.nih.gov/sra/), the accession number for our submission is: PRJNA645038.

## Abbreviations

ABC: ATP-binding cassette
ABI: ABA insensitive
AOC: Allene oxide cyclase
AOS: Allene oxide synthase
AP2: Apetala-2
ARF: Auxin response factor
bHLH: Basic helix-loop-helix
bZIP: Basic leucine zipper
CAM: Calmodulin
CDPK: Calcium-dependent protein kinase
CAT: Catalase
CML: Calmodulin like
CPK: Calmodulin-domain protein kinase
COMT: Caffeic acid O-methyltransferase-like
DEGs: Differentially expressed genes
EREBP: Ethylene response element binding protein
ERF: Ethylene response factor
FDR: False discovery rate
GH: Glycoside hydrolases
GST: Glutathione S-transferase
GT: Glycosyltransferases
HB: Homeobox
HKT: High-affinity K^+^ transporter
HSF: Heat shock factor
LLD: Legume-lectin domain
MAPK: Mitogen-activated protein kinase
MDA: Malondialdehyde
MYB: Myeloblastosis
NCED: 9-cis-epoxycarotenoid dioxygenase
NHX: Na^+^/K^+^ exchanger
PAL: Phenylalanine-ammonia lyase
PERK: Proline extensin like
PP2C: Protein phosphatase 2C
POD: Peroxidase
ROS: Reactive oxygen species
SKP: Sphase-associated kinase protein kinases
SOD: Superoxide dismutase
TCA: Tricarboxylic
UGE: UDP-glucose 4-epimerase
WAK: S-locus glycoprotein like and wall associated receptor kinase
XTH: Xyloglucan endotransglucosylase
