# Supplementary material for: Comprehensive transcriptional analysis reveals salt stress-regulated key pathways, hub genes and time-specific responsive gene categories in common bermudagrass (Cynodon dactylon (L.) Pers.) roots
Source: BMC Plant Biol. 2021 Apr 10;21:175. doi: 10.1186/s12870-021-02939-1 (PMC8035780; doi:10.1186/s12870-021-02939-1)
Supplement: Supplementary file 1 — Additional file 1: Fig. S1. Example of down-regulated gene categories following salt treatment at different time points using PageMan. Fig. S2. Examples of genes commonly up-regulated at three-time points or specifically up-regulated at 1 h. Gene IDs and are indicated at the right side of each heat map. Fig. S3. Protein metabolism regulated following salt treatment at different time points using PageMan. SvsCK1h (left column), SvsCK6h (middle column) and SvsCK24h (right column). Fig. S4. Example of gene categories specifically up-regulated following 1 h salt treatment using PageMan. SvsCK1h (left column), SvsCK6h (middle column) and SvsCK24h (right column). Fig. S5. Transcription factors up-regulated following salt treatment at different time points using PageMan. Fig. S6. The complete view of enriched gene categories using pageman analysis. SvsCK1h (left column), SvsCK6h (middle column) and SvsCK24h (right column). Fig. S7. Relative expression levels of hub genes in the lavenderblush2 and brown4 modules. a-c, Hub genes from lavenderblush2 module. d-h, Hub genes from brown4 module. Relative expression level is presented as the mean ± SD of three replicates at each time point. Expression levels of hub genes were calculated using the 2−△CT approach using Actin2 as the reference gene. Table S1. The list of DEGs genes under different time point respectively and the fold change of DEGs for STEM package. Table S2. The list of time-specific enriched sub-bins of DEGs using MapMan systems. The genes from different enriched categories at each time point are given in log2 scale, and can be distinguished by the column headers. Table S3. The list of DEGs commonly regulated at all three time points. The genes from different enriched categories at each time point is given in log2 scale. Table S4. The physiological index used for WGCNA analysis and hub genes involved in lavenderblush2 and brown4 modules. Table S5. The primes of hub genes used for RT-qPCR. [file 12870_2021_2939_MOESM1_ESM.pdf]

Additional File 1

Click here to access/download
Supplementary Material
Fig. S1.tif

Additional File 2

Click here to access/download
Supplementary Material
Fig. S2.tif

Additional File 3

Click here to access/download
Supplementary Material
Fig. S3.tif

Additional File 4

Click here to access/download
Supplementary Material
Fig. S4.tif

Additional File 5

Click here to access/download
Supplementary Material
Fig. S5.tif

Additional File 6

Click here to access/download
Supplementary Material
Fig. S6.TIF

Additional File 7

Click here to access/download
Supplementary Material
Fig. S7.tif

Additional File 8

Click here to access/download
Supplementary Material
Table S1 .xls

Additional File 9

Click here to access/download
Supplementary Material
Table S2.xlsx

Additional File 10

Click here to access/download

Supplementary Material

Table S3.xlsx

Additional File 11

Click here to access/download
Supplementary Material
Table S4.xlsx

Additional File 12

Click here to access/download

Supplementary Material

Table S5.xlsx
